# Performance of Cleveland, Mehta, and Simplified Renal Index scores for predicting dialysis-requiring acute kidney injury after aortic *vs.* non-aortic cardiac surgery (2006–2023, 6160 patients)

**DOI:** 10.1080/0886022X.2025.2592437

**Published:** 2025-11-26

**Authors:** Christopher Pak-To Lee, Karen Hoi-Ling Ng, Gordon Yuk-Sang Choi, Wai-Tat Wong, Henry Man Kin Wong, Kwok-Ming Ho, Lowell Ling, Randolph Hung-Leung Wong

**Affiliations:** ^a^ Department of Intensive Care, Prince of Wales Hospital, Hong Kong SAR, China; ^b^ Department of Anaesthesia and Intensive Care, The Chinese University of Hong Kong, Hong Kong SAR, China; ^c^ Division of Cardiothoracic Surgery, Prince of Wales Hospital, The Chinese University of Hong Kong, Hong Kong SAR, China; ^d^ Department of Anaesthesia, Pain and Perioperative Medicine, Prince of Wales Hospital, Hong Kong SAR, China

**Keywords:** Acute kidney injury, cardiac surgery, critical illness, renal replacement therapy, clinical score, risk prediction

## Abstract

Acute kidney injury requiring renal replacement therapy (RRT-AKI) is a serious complication after cardiac surgery, especially in aortic procedures. Validated risk models, such as the Cleveland Clinic Score, Mehta tool, and Simplified Renal Index (SRI) are widely applied, but their performance in predicting after aortic versus non-aortic cardiac surgery remains uncertain. We conducted a retrospective cohort study of 6,160 patients undergoing cardiac surgery (1,002 aortic and 5,158 non-aortic) (2006–2023) at a teaching hospital in Hong Kong. Predictive performance of the risk scores were assessed using the area under the receiver operating characteristic curve (AUC). Differences in AUC were assessed using DeLong’s test. Hosmer-Lemeshow goodness-of-fit test was used to assess calibration. In this cohort, 3.2% developed RRT-AKI. RRT-AKI was more common after aortic surgery than non-aortic surgery (6.7 *vs.* 2.5%; *p* = <.001). Thirty-day mortality reached 40% in patients with acute kidney injury requiring renal replacement therapy after cardiac surgery. In the non-aortic surgery cohort, the three scores only showed moderate discriminatory power (worse than original cohorts) and good calibration: 0.77 (95% CI, 0.72–0.81) and *p* = .62 (Cleveland); 0.75 (95% CI, 0.70–0.80) and *p* = .10 (Mehta); and 0.75 (95% CI, 0.71–0.79) and *p* = .65 (SRI). In the aortic surgery cohort, there was only moderate discriminatory power and good calibration: AUC 0.70 (95% CI, 0.63–0.77) and *p* = .57 (Cleveland); 0.67 (95% CI, 0.59–0.74) and *p* = .67 (Mehta); 0.65 (95% CI, 0.58–0.72) and *p* = .79 (SRI). Currently established risk scores only have moderate discriminatory power (range of AUC 0.65–0.77) to predict RRT-AKI after both cardiac and aortic surgery.

## Graphical abstract

**Figure:**
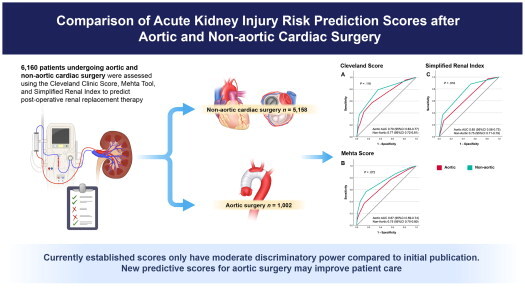

## Background

Cardiac surgery-associated acute kidney injury (CS-AKI) is one of the most serious complications of cardiac surgery with major consequences, such as acute kidney injury requiring renal replacement therapy [1]. It is associated with increased postoperative mortality, longer intensive care unit (ICU) and hospital length of stays [2–4], as well as increased morbidity extending beyond the hospital stay, such as development of chronic kidney disease [5] and adverse cardiovascular events like heart failure [6].

The overall incidence of CS-AKI ranges from 20 to 30% [7]. However, data show that different types of cardiac surgery are associated with different risks. One study reported a lower incidence of AKI rate in patients undergoing coronary artery bypass graft (CABG) surgery (19.0%) compared to those who underwent valve surgery (27.5%) or aortic surgery (29.0%) [8]. Another study of thoracic aortic surgery specifically showed a remarkably high incidence of AKI of 54%, with 11% needing renal replacement therapy [9], rates which are well beyond that described with other cardiac surgical procedures.

The Cleveland Clinic Score [10], Mehta tool [11], and the Simplified Renal Index (SRI) [12], are three validated clinical risk scores to predict the need for postoperative RRT in patients undergoing cardiac surgery. Although all three predictive scores include the type of surgery as risk variables, none specifically distinguish between aortic and non-aortic cardiac surgical procedures, nor describe the relative proportions of different procedures in their original derivation cohort. It is uncertain whether these scores have the same predictive performance for aortic procedures compared to non-aortic procedures.

Given the higher prevalence of AKI seen after aortic surgery, we hypothesized that the predictive performance of these three commonly used scores would be weaker for patients undergoing aortic surgery compared to non-aortic cardiac surgery. Therefore, we undertook a retrospective cohort study to describe the characteristics of patients who require postoperative RRT after aortic and non-aortic cardiac surgery at our institution, and to compare the predictive performance of these three scores when applied to these two groups of patients.

## Methods

### Study design and population

This was a retrospective study of prospectively collected data on adult patients (age ≥ 18 years) who underwent cardiac surgery, including coronary artery bypass grafting (CABG), mitral or aortic valve surgery, and aortic surgery with cardiopulmonary bypass (CPB) between 1 January 2006 and 31 December 2023 at the Prince of Wales Hospital, Hong Kong SAR in China. All patients were included if all three post-operative AKI scores could be calculated from collected data. Exclusion criteria were based on the exclusion criteria of the Cleveland, Mehta, and SRI scores, which included pre-operative use of either renal replacement therapy, extracorporeal membrane oxygenation (ECMO), mechanical ventilation, or tracheostomy. If patients underwent more than one cardiac surgery with CPB during the study period, only data from the first index cardiac surgery were assessed. The study was approved by the Joint Chinese University of Hong Kong–New Territories East Cluster Clinical Research Ethics Committee (2024.412).

### Study site characteristics

Prince of Wales Hospital is a teaching hospital and a regional tertiary cardiothoracic surgery referral center. Prince of Wales ICU is a 28-bed, level III multidisciplinary unit as defined by the College of Intensive Care Medicine of Australia and New Zealand (CICM) [13]. A team of intensive care specialists provides 24-h specialist care to over 2000 critically ill patients each year. The 2018 APACHE standardized mortality ratio of the unit was 0.67 [14]. All patients who undergo cardiopulmonary bypass are routinely managed in the ICU in the immediate postoperative period and usually discharged to the cardiothoracic high dependency unit within 24 h after extubation. In our center, RRT is initiated for standard indications, such as uremia, volume overload, or refractory biochemical abnormalities [15].

### Data collection and outcomes

Patients were identified from a prospective computerized database called the Dendrite clinical system (Dendrite Clinical System, Ltd, Oxford, England, United Kingdom). The Dendrite Cardiac Surgery data collection system was established in 2005 at our hospital. It collects clinically relevant data, such as preoperative medical history and postoperative complications for patients who undergo cardiac surgery. We collected all variables required to calculate each of the three predictive scores.

Post-operative RRT was defined as initiation of any form of RRT after the index operation during the same hospital stay. Mortality was defined as hospital mortality within 30 days of the index operation.

### Statistical analysis

Descriptive statistics for categorical variables are expressed as frequency (percentage), while continuous variables are expressed as mean ± standard deviation (*SD*). Categorical variables were compared between cohorts with and without postoperative RRT using the Chi-square test or Fisher’s exact test, when applicable. Continuous variables were compared using an independent sample *t*-test or Wilcoxon rank-sum test, when applicable. Multivariate logistic regression was used to identify risk factors associated with post-operative RRT.

The performance of each clinical score in predicting acute kidney injury requiring renal replacement therapy after cardiac surgery was assessed using the area under the receiver operating characteristic curve (AUC). Hosmer-Lemeshow goodness-of-fit tests were used to perform calibration of each clinical score, with logistic regression models used to calculate the predicted probabilities for the need for RRT. The original AKI risk prediction scores provided risk stratification by categorizing score ranges instead of defining specific thresholds for the binary outcome of acute kidney injury requiring renal replacement therapy after cardiac surgery. Therefore, to evaluate clinical predictive performance of different scores, Youden’s index was used to identify optimal thresholds to calculate sensitivity, specificity, positive predictive value, negative predictive value. Good discrimination and calibration refer to AUC > 0.80 and *p*-value > 0.05, respectively. The non-parametric DeLong test was used to make pairwise comparisons of AUCs. Two-sided statistical tests were performed, with a significant level set at 0.05 for statistical significance. Statistical analysis was performed using SPSS statistics for Windows (Version 29.0.1.0., IBM Corp, Armonk, NY, USA).

## Results

### Cohort characteristics

**Figure 1.**
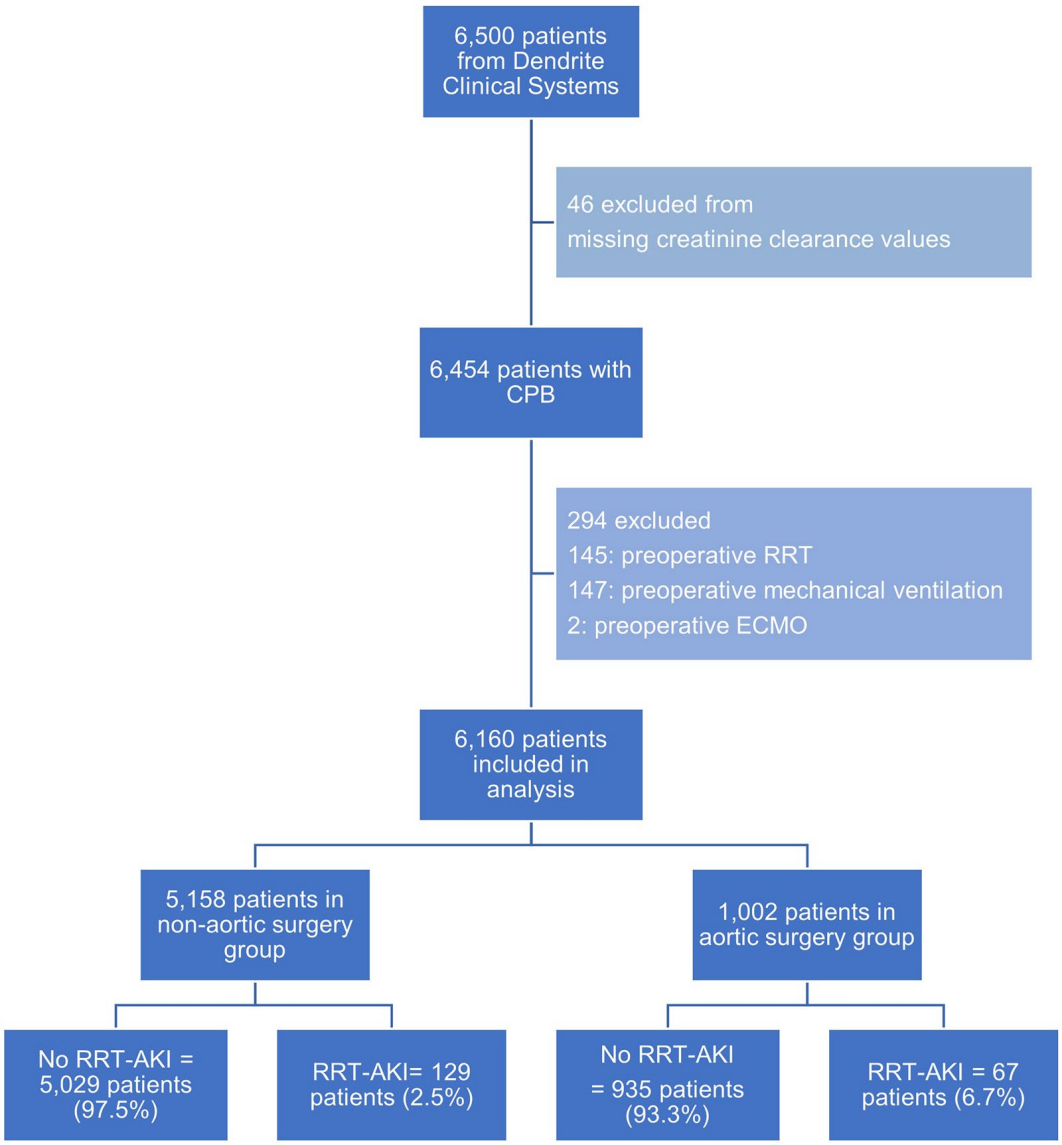
CONSORT flow diagram of patient population selection. CPB: cardiopulmonary bypass; ECMO: extracorporeal membrane oxygenation; RRT-AKI: acute kidney injury requiring renal replacement therapy after cardiac surgery.

A total of 6,500 patients who underwent cardiopulmonary bypass for cardiac surgery were identified from the Dendrite database. After excluding cases with missing plasma creatinine clearance values (*n* = 46), pre-operative RRT (*n* = 145), mechanical ventilation (*n* = 147), and ECMO (*n* = 2), 6,160 patients were included in the validation cohort (Figure 1). Among the 6,160 eligible patients, 1,002 and 5,158 patients underwent aortic and non-aortic surgeries, respectively.

The baseline characteristics of our study cohort are described in Table 1. Overall, acute kidney injury requiring renal replacement therapy after cardiac surgery occurred in 196 (3.2%) patients. The incidence of acute kidney injury requiring renal replacement therapy after cardiac surgery was more than 2-fold higher after aortic surgery (6.7%) compared to non-aortic cardiac surgery (2.5%) (*p* < .001). These rates remained stable over the study period (*p* = .15). Patients with acute kidney injury requiring renal replacement therapy after cardiac surgery were statistically more likely to have pre-operative impaired renal function, hypertension, diabetes mellitus requiring insulin, chronic obstructive pulmonary disease, and congestive heart failure, or use of pre-operative intra-aortic balloon pump (IABP), or cardiogenic shock. In terms of surgical factors, patients who had previous cardiac surgery, or were undergoing aortic surgery or emergency surgery were also more likely to develop acute kidney injury requiring renal replacement therapy after cardiac surgery. The 30-day mortality was higher for patients who developed acute kidney injury requiring renal replacement therapy after cardiac surgery (40.3%) compared to those who did not (2.2%) (*p* < .001).

**Table 1.** Demographics and characteristics of patients who developed acute kidney injury requiring renal replacement therapy after cardiac surgery (RRT-AKI) compared to patients who did not (non RRT-AKI).

	Non RRT-AKI *n* = 5,964 (%)	RRT-AKI *n* = 196 (%)	*p*-Value
Demographics			
Age, median (IQR), years	62 (55–69)	64 (56–70)	.049
Female	1,731 (29.0)	68 (34.7)	.086
Preoperative kidney function			
Serum creatinine, median (IQR), μmol/L	88.4 (79.6–114.9)	123.8 (88.4–176.8)	<.001
GFR^a^ ≤30 mL/min	249 (4.2)	57 (29.1)	<.001
Comorbidities			
Hypertension	3,745 (62.8)	153 (78.1)	<.001
Diabetes mellitus on oral therapy	1,210 (20.3)	37 (18.9)	.63
Diabetes mellitus on insulin therapy	308 (5.2)	21 (10.7)	<.001
COPD	510 (8.6)	25 (12.8)	.040
Stroke with residual deficit	112 (1.9)	7 (3.6)	.11
Cardiac status			
Recent MI ≤ 30 days	578 (9.7)	20 (10.2)	.81
CHF	834 (14.0)	57 (29.1)	<.001
LVEF ≤ 35%	321 (5.4)	16 (8.2)	.092
Preoperative IABP	171 (2.9)	11 (5.6)	.026
Cardiogenic shock	148 (2.5)	20 (10.2)	<.001
Previous cardiac surgery	472 (7.9)	30 (15.3)	<.001
Surgical details			
Aortic surgery	935 (15.7)	67 (34.2)	<.001
Emergency surgery	556 (9.3)	59 (30.1)	<.001
Time period			
2006–2011	1,791 (30.0)	51 (26.0)	0.15
2012–2017	1,950 (32.7)	77 (39.3)	
2018–2023	2,223 (37.2)	68 (34.7)	
Outcome			
30-day mortality	130 (2.2)	79 (40.3)	<.001

RRT-AKI: acute kidney injury requiring renal replacement therapy after cardiac surgery; GFR: glomerular filtration rate; COPD: chronic obstructive pulmonary disease; CVA: cerebrovascular accident; MI: myocardial infarction; CHF: congestive heart failure; LVEF: left ventricular ejection fraction; IABP: intra-aortic balloon pump.

^a^ Data for GFR are estimated using the Cockcroft-Gault equation.

### Factors associated with acute kidney injury requiring renal replacement therapy after cardiac surgery

**Figure 2.**
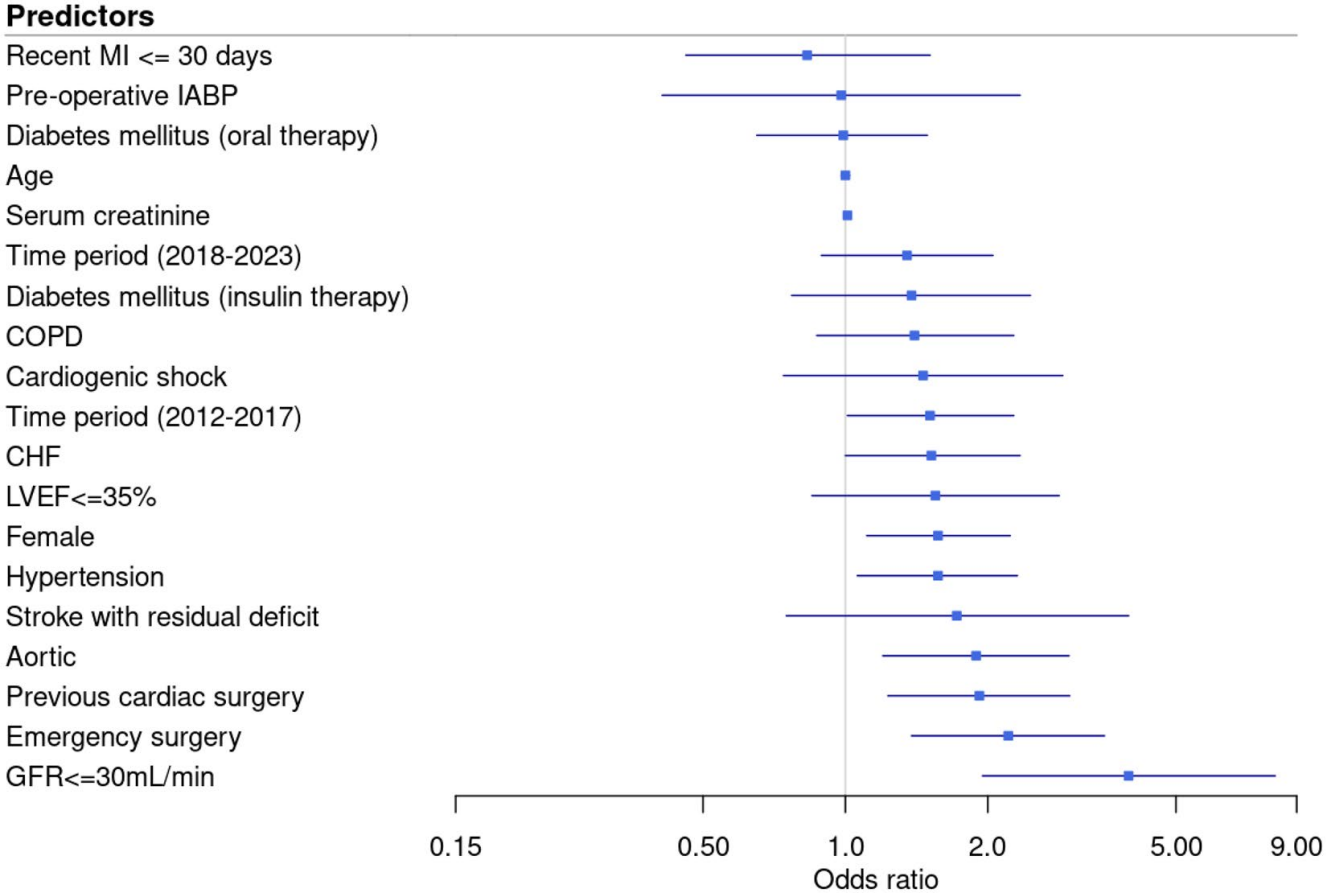
Forest plot of risk factors identified on multivariate analysis associated with acute kidney injury requiring renal replacement therapy after cardiac surgery. Y axis, risk factor. X axis, adjusted odds ratio with 95% confidence intervals. ^a^Data for GFR are estimated using the Cockcroft-Gault equation. OR: odds ratio; GFR: glomerular filtration rate; COPD: chronic obstructive pulmonary disease; MI: myocardial infarction; CHF: congestive heart failure; LVEF: left ventricular ejection fraction; IABP: intra-aortic balloon pump.

On multivariate analysis, the risk factors that were associated with acute kidney injury requiring renal replacement therapy after cardiac surgery included preexisting renal impairment, emergency surgery, previous cardiac surgery, aortic surgery, hypertension, and female sex (Table 2 and Figure 2).

**Table 2.** Multivariate logistic regression model of risk factors associated with acute kidney injury requiring renal replacement therapy after cardiac surgery (RRT-AKI).

	Adjusted OR (95% CI)	*p*-Value
Demographics		
Age	1.00 (0.98–1.02)	.85
Female	1.57 (1.11–2.23)	.012
Preoperative kidney function		
Serum creatinine	1.01 (1.01–1.01)	<.001
GFR^a^ ≤30 mL/min	3.97 (1.95–8.10)	<.001
Comorbidities		
Hypertension	1.57 (1.06–2.31)	.023
Diabetes mellitus (oral therapy)	0.99 (0.65–1.49)	.95
Diabetes mellitus (insulin therapy)	1.38 (0.77–2.46)	.28
COPD	1.40 (0.87–2.27)	.17
Stroke with residual deficit	1.72 (0.75–3.97)	.20
Cardiac status		
Recent MI ≤ 30 days	0.83 (0.46–1.51)	.55
CHF	1.52 (1.0–2.34)	.05
LVEF ≤ 35%	1.55 (0.85–2.83)	.15
Pre-operative IABP^i^	0.98 (0.41–2.34)	.96
Cardiogenic shock	1.46 (0.74–2.88)	.28
Previous cardiac surgery	1.92 (1.23–2.98)	.004
Surgical details		
Aortic surgery	1.89 (1.20–2.97)	.006
Emergency surgery	2.21 (1.38–3.53)	<.001
Time period		
2006–2011		.13
2012–2017	1.51 (1.01–2.27)	
2018–2023	1.35 (0.89–2.05)	

OR: odds ratio; GFR: glomerular filtration rate; COPD: chronic obstructive pulmonary disease; MI: myocardial infarction; CHF: congestive heart failure; LVEF: left ventricular ejection fraction; IABP: intra-aortic balloon pump.

^a^ Data for GFR are estimated using the Cockcroft-Gault equation.

### Prediction of acute kidney injury requiring renal replacement therapy after cardiac surgery

**Figure 3.**
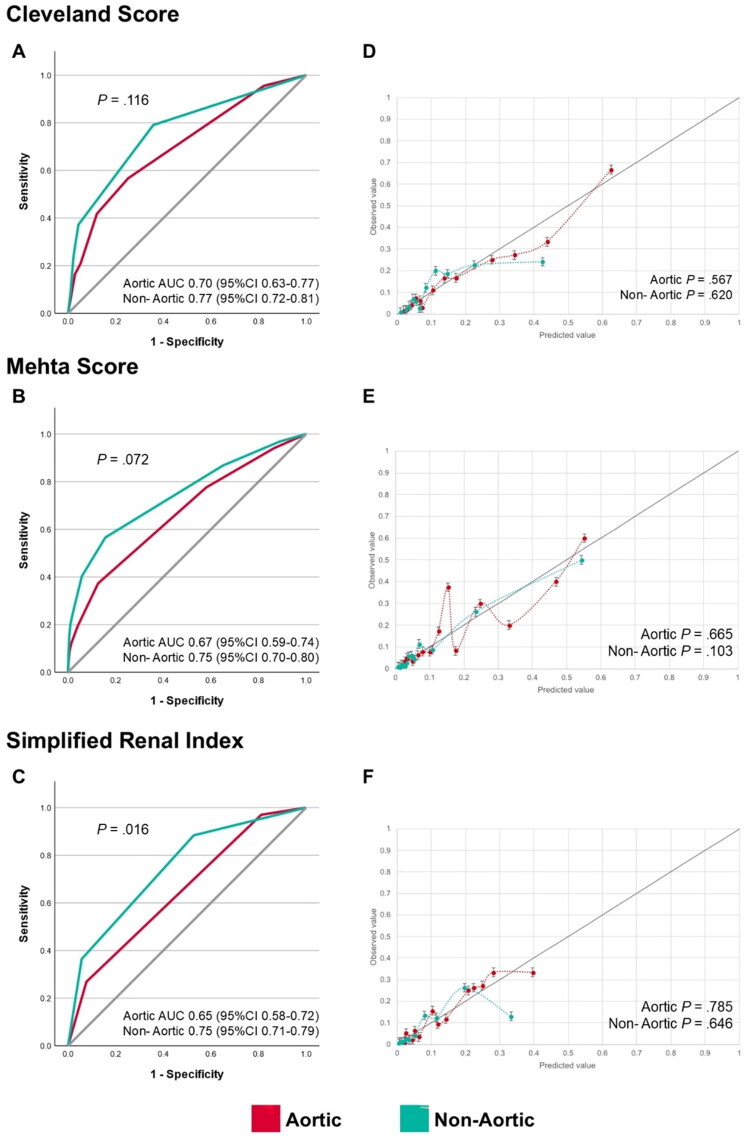
Performance-based classification of AKI risk scores (Cleveland Clinic Score, Mehta tool, Simplified Renal Index). Outcomes: dialysis-requiring AKI (RRT-AKI). Receiver operating characteristic curves for discriminating need for acute kidney injury requiring renal replacement therapy after cardiac surgery of (A) Cleveland score, (B) Mehta score, and (C) Simplified Renal Index in aortic and non-aortic surgery. Calibration curves of observed and predicted need for acute kidney injury requiring renal replacement therapy after cardiac surgery for (D) Cleveland score, (E) Mehta score, and (F) Simplified Renal Index in aortic and non-aortic surgery. AUC: area under receiver operating curve.

The ability of all three clinical scores to discriminate risk of acute kidney injury requiring renal replacement therapy after cardiac surgery was fair (AUC 0.65 to 0.77) for both aortic and non-aortic cardiac surgery (Figures 3(A–C)). Paired Delong’s test to compare the performance of scores in the aortic cohort showed no statistically significant difference between scores (Cleveland *vs.* Mehta, *p* = .49; Cleveland *vs.* SRI, *p* = .31; and Mehta *vs.* SRI, *p* = .77). Similarly, paired Delong’s test to compare performance amongst the three predictive scores in the non-aortic cohort showed no statistically significant difference (Cleveland *vs.* Mehta, *p* = .55; Cleveland *vs.* SRI, *p* = .58; Mehta *vs.* SRI, *p* = .94). Although the predictive performance of each score was numerically higher in the non-aortic cohorts than aortic cohorts, this difference was only significant for the SRI score (*p* = .016). AUC of the three scores in predicting RRT for aortic and non-aortic cardiac surgery was variable but did not appear to consistently deteriorate over time between 2007 to 2023 (see Additional File 1 eFigures 1–3).

The calibration of the three renal risk scores was acceptable when applied to both the aortic surgery cohort (Cleveland Clinic score: *p* = .57; Mehta tool: *p* = .67; and SRI: *p* = .79) (Figures 3(D–F)) and the non-aortic cohort (Cleveland Clinic score: *p* = .62; Mehta tool: *p* = .10; and SRI: *p* = .65). An Additional File Supplementary Tables (eTables 1–3) describes the frequency of acute kidney injury requiring renal replacement therapy after cardiac surgery by risk category of each score (see Additional File 1).

The sensitivity, specificity, PPV, and NPV of the three predictive scores are described in Table 3. Within the aortic cohort, the Cleveland Clinic score had the highest sensitivity and specificity, whereas the Mehta tool had the lowest sensitivity and specificity. For the non-aortic cohort, no score consistently showed better sensitivity and specificity. The PPV was low for all scores, which may be related to the low prevalence of acute kidney injury requiring renal replacement therapy after cardiac surgery in this study. The NPV was high for all scores except for when the Mehta tool was applied to aortic cohort, where it decreased to 0.82 (aortic cohort) from 0.99 (non-aortic cohort).

**Table 3.** Predictive performance of AKI scores.

	Cleveland			Mehta			Simplified Renal Index		
	Aortic	Non-aortic	Overall	Aortic	Non-aortic	Overall	Aortic	Non-aortic	Overall
Sensitivity (95% CI)	0.78 (0.75–0.80)	0.65 (0.64–0.66)	0.73 (0.72–0.75)	0.57 (0.54–0.60)	0.71 (0.69–0.72)	0.58 (0.57–0.59)	0.60 (0.57–0.63)	0.69 (0.68–0.70)	0.66 (0.65–0.67)
Specificity (95% CI)	0.56 (0.53–0.59)	0.83 (0.82–0.84)	0.76 (0.74–0.77)	0.15 (0.12–0.17)	0.70 (0.69–0.72)	0.77 (0.76–0.78)	0.63 (0.60–0.66)	0.80 (0.79–0.81)	0.78 (0.77–0.79)
PPV (95% CI)	0.11 (0.09–0.13)	0.09 (0.08–0.10)	0.09 (0.08–0.10)	0.05 (0.03–0.06)	0.06 (0.05–0.06)	0.08 (0.07–0.08)	0.10 (0.09–0.12)	0.08 (0.08–0.09)	0.09 (0.08–0.10)
NPV (95% CI)	0.97 (0.96–0.98)	0.99 (0.98–0.99)	0.99 (0.98–0.99)	0.82 (0.80–0.85)	0.99 (0.98–0.99)	0.98 (0.98–0.99)	0.96 (0.94–0.97)	0.99 (0.98–0.99)	0.99 (0.98–0.99)

CI: confidence interval; NPV: negative predictive value; PPV: positive predictive value.

The specificity, sensitivity, NPV, and PPV results were based on the thresholds identified by cut points generating the highest Youden indexes. For the overall cohort, the thresholds used for the Cleveland, Mehta, and SRI scores were 3.5, 18, and 2.5, respectively. The thresholds for the Cleveland, Mehta, and SRI scores in the aortic subgroup were 4.5, 18.5, and 2.5, respectively; whilst the thresholds used for the Cleveland, Mehta, and SRI scores in the non-aortic subgroup were 3.5, 16.5, and 2.5, respectively.

## Discussion

In this retrospective cohort study of 6,160 patients undergoing aortic and non-aortic cardiac surgery, we found that acute kidney injury requiring renal replacement therapy after cardiac surgery occurred in 3.2% of patients. Aortic surgery was associated with acute kidney injury requiring renal replacement therapy after cardiac surgery even after adjustment for other covariates. The Cleveland Clinic, Mehta tool, and SRI risk scores only had moderate discriminatory power to predict acute kidney injury requiring renal replacement therapy after cardiac surgery in aortic and non-aortic surgery. Only SRI score showed a difference in predictive performance for post-operative RRT between aortic and non-aortic surgery.

Consistent with international literature, we found that acute kidney injury requiring renal replacement therapy after cardiac surgery after cardiac surgery was an uncommon event at 3.2%. Nevertheless, this was higher than observed rates in the derivation cohorts of the Cleveland Clinic Score (1.7%), SRI (1.3–2.2%), and Mehta Tool (1.4%) [10–12]. The reported range of acute kidney injury requiring renal replacement therapy after cardiac surgery after cardiac surgery in the current literature ranges from 1–5%, likely as a result of variations in casemix [16]. For example, a large cohort including only isolated coronary artery surgery reported a lower incidence of acute kidney injury requiring renal replacement therapy after cardiac surgery of 1.3% [17]. Another cohort which included both coronary artery surgery as well as valvular surgery and congenital heart surgery showed much higher rate of 7.9% [18]. A large meta-analysis which combined 47 cohorts reported a pooled rate of 2.1% [19]. Major validation cohorts, such as Englberger et al. 2010, Kiers et al. 2013, and Jiang et al. 2017 have also shown similar rates of acute kidney injury requiring renal replacement therapy after cardiac surgery of 1.1–2.1%. In our cohort, 10% of patients underwent emergency cardiac surgery, which may explain the relatively higher rate of acute kidney injury requiring renal replacement therapy after cardiac surgery. Although the exact proportion of aortic cases were not reported in the original derivation cohorts, the relatively high volume of aortic surgery in our center may also explain why the rate of acute kidney injury requiring renal replacement therapy after cardiac surgery was higher in our cohort. On multivariate analysis (Table 2), we identified emergency surgery and aortic surgery as being consistent high risk predictors for acute kidney injury requiring renal replacement therapy after cardiac surgery. We also identified female sex as a predictor for acute kidney injury requiring renal replacement therapy after cardiac surgery which is consistent with literature reporting higher rates of AKI after cardiac surgery in women [20,21].

The increased incidence of AKI after aortic surgery compared to non-aortic cardiac surgery is well reported in the literature [22,23]. There are several biological reasons for why aortic surgery may be associated with more severe acute kidney injury [24]. Pathophysiological mechanisms after acute aortic dissection include preoperative hypoperfusion of the kidneys [25] and hemodynamic instability [26,27]. In addition, there are differences in surgical technique. These include longer cardiopulmonary bypass time which is associated with increased risk of AKI when compared to non-aortic surgery [28]. The need for hypothermic circulatory arrest during aortic surgery may also result in systemic inflammatory response syndrome and ischemia-reperfusion injury to the kidneys. These mechanisms may act as a second hit in an already diseased kidney [29]. Our multivariate analysis confirmed that, even after accounting for emergent cases, aortic surgery was still associated with acute kidney injury requiring renal replacement therapy after cardiac surgery.

Overall, we found that the three predictive risk scores only had moderate predictive performance to identify patients at risk of post-operative RRT after aortic and non-aortic cardiac surgery. Amongst the three scores, the Cleveland Clinic score showed the highest AUC for both the aortic and non-aortic groups; however, it was still only moderately predictive, with an AUC of 0.70 (aortic) and 0.77 (non-aortic). This is lower than the rates described in the original studies. It is important to note that studies on the predictive performance of these scores have varied in the endpoint chosen varying from acute kidney injury as defined by increase in serum creatinine, or by postoperative RRT as used in our study [30–33]. However, even when comparing specifically for acute kidney injury requiring renal replacement therapy after cardiac surgery, there is variation in performance described. A large cohort study of more than 12,000 patients in the United States by Englberger et al. in 2010 similarly found good discriminatory power close to the original cohorts with AUCs in the range of 0.79–0.86 for the three scores [30]. Another smaller study of 1,409 patients in the Netherlands by Kiers et al. in 2013 also found better discriminatory power than compared to our cohort, with AUC in the range of 0.85–0.93 for the three scores [31]. However, studies in Chinese patients, including Zhang et al. 2022 and Jiang et al. 2017 found AUCs lower than the original cohorts with a range of 0.62–0.70 over the three scores [32,33], which raises the possibility that different models may be more suitable to apply to different local populations. Differences in demographics and preoperative co-morbidities amongst patients, surgical selection may result in the same risk factor carrying different weights in different populations. Consequently, it may be important to incorporate healthcare organizational factors in future models to account for regional variations and maximize predictive performance across different settings and populations. Our findings may inform future meta-analyses comparing AKI prediction scores across populations, particularly in Asian cohorts. Whilst the original scores were based on older data sets from 2005 to 2007, our study found that their predictive performance have consistently remained moderate without temporal reduction for aortic and non-aortic surgery in our population since their publication (see Additional File 1 eFigures 1–3). Nevertheless, deterioration in risk prediction performance over time have been reported in percutaneous interventions such as transcatheter aortic valve implantation [34]. Although contemporary predictive scores have been published, their utility remains uncertain due to limitations such as small development data sets of AKICS score [35] which was derived from only 603 patients, or have been single center cohorts [36,37] that lack external validation.

Although only SRI showed reduced predictive performance in aortic surgery when compared to use in non-aortic cardiac surgery, the trends were similar for Cleveland and Mehta Tool. Given the overall low event rate in our cohort, a larger sample size in the future may be useful to confirm such findings or detect a difference, as the size of our current cohort was smaller than the original derivation cohorts. There are several reasons why current scores may underperform for aortic surgery. The current scores examined were developed in cohorts that did not include aortic surgery [11] or only included small proportions of patients undergoing aortic surgery [10,12]. There have been no studies as of yet specifically examining whether these scores perform differently in aortic compared to non-aortic surgery with respect to acute kidney injury requiring renal replacement therapy after cardiac surgery. The additional pathophysiological mechanisms described above may not be adequately captured by the original scoring criteria which do not include aortic-specific risk factors such as duration of renal ischemia [38], and incorporating these into future scoring criteria may improve risk prediction in this setting. At the moment, the only scoring system specifically for aortic surgery [39] available predicts AKI as defined by RIFLE criteria [40]. Similar to our study, it found that the three scores had suboptimal performance for predicting AKI after aortic surgery. However, its applicability is limited by not predicting specifically for acute kidney injury requiring renal replacement therapy after cardiac surgery, a small derivation sample size, and lack of external validation.

This study contributes to the literature by validating three widely used scores (Cleveland Clinic Score, Mehta Tool, and Simplified Renal Index) in patients undergoing both aortic and non-aortic cardiac surgery. There are several clinical implications of our findings. Firstly, we found that there has been a reduction in the performance of the three scores compared to when they were first published. Centers should consider their own case-mix before applying these scores for clinical use and be aware that there may be reduced predictive power over time. This limits their clinical utility, particularly for centers where there is a high proportion of emergency or aortic surgery in the overall case-load. Future research to improve this area of practice could include a new re-calibrated score or devising a specific score for aortic surgery. To our knowledge, there are no well-established clinical risk scores that are specifically focused on predicting postoperative RRT after aortic surgery. The current SRI and Cleveland scores can allow non-CABG/valve surgery to be coded separately which were both significantly associated with an increased risk of RRT in our patients (with an OR >2.7). Therefore, including detailed information specific to aortic surgery, such as operative time, hypothermic circulatory arrest time, may further improve their predictive performance. Additionally, apart from relying on scoring systems and clinical criteria, it may be that other ways to predict risk may be more accurate in future, such as with use of novel biomarkers of renal injury both pre and post-surgery [41,42], either standalone or integrated with other data in machine learning models [43]. Epidemiological data from other countries have also shown a trend of increased surgery rates for aortic dissection, highlighting the importance of accurate risk prediction in aortic surgery [44].

Our study has some limitations. Firstly, the data is limited to a single tertiary center and reflects our institution’s practice and the specific characteristics of our patient population. There may be differences in perioperative management and technique leading to different rates of kidney injury in other centers, which may affect the performance of these three clinical risk scores. For example, the majority of our patients underwent cardiac surgery with CPB at our center (94.6%). In contrast, patients who underwent off-pump CABG were also included in the Cleveland Clinic score derivation cohort [10]. Secondly, we measured postoperative RRT but did not record the exact reason for RRT nor the rates of severe AKI (KDIGO stage 3 not requiring RRT). Third, differences in practice of RRT may differ across centers since the optimal timing to initiate RRT is controversial [45,46], and it is known that there are variations in practice among different centers [47,48]. Although clinical decision for initiation of RRT in our center is based on commonly accepted criteria, it is still subject to individual variations in clinical practice. Finally, we did not collect data on postoperative outcomes beyond 30-day mortality, such as the incidence of chronic kidney disease or need for long-term dialysis. It has been reported that post-operative AKI is associated with long term renal dysfunction, so it may be significant even if not requiring RRT [49–51]. These would be important patient-centered outcomes to address in future prospective studies.

## Conclusion

In summary, need for post-operative RRT remains an uncommon event after cardiac surgery but is much more common after aortic surgery. Currently established risk scores only have moderate discriminatory power to predict need for RRT after both cardiac and aortic surgery. Given our findings that patients who develop acute kidney injury requiring renal replacement therapy after cardiac surgery have a significantly higher 30 day mortality, improvement is important for future risk model development. Our findings are relevant for future meta-analyses of AKI risk models, machine learning model development, and the design of aortic surgery–specific prediction scores.

## Supplementary Material

Additional_file_1_v3 - Clean file.docx

## Data Availability

All data supporting the findings of this study are available within the paper and its Additional Files. Deidentified individual participant data can be available upon reasonable request sent to the corresponding author and approval from ethics committee.

## References

[1] Wang Y, Bellomo R. Cardiac surgery-associated acute kidney injury: risk factors, pathophysiology and treatment. Nat Rev Nephrol. 2017;13(11):697–711. doi: 10.1038/nrneph.2017.119. 28869251

[2] Loef BG, Epema AH, Smilde TD, et al. Immediate postoperative renal function deterioration in cardiac surgical patients predicts in-hospital mortality and long-term survival. J Am Soc Nephrol. 2005;16(1):195–200. doi: 10.1681/ASN.2003100875. 15563558

[3] Kumar AB, Suneja M, Bayman EO, et al. Association between postoperative acute kidney injury and duration of cardiopulmonary bypass: a meta-analysis. J Cardiothorac Vasc Anesth. 2012;26(1):64–69. doi: 10.1053/j.jvca.2011.07.007. 21924633

[4] Mao H, Katz N, Ariyanon W, et al. Cardiac surgery-associated acute kidney injury. Cardiorenal Med. 2013;3(3):178–199. doi: 10.1159/000353134. 24454314 PMC3884176

[5] Cho JS, Shim JK, Lee S, et al. Chronic progression of cardiac surgery associated acute kidney injury: intermediary role of acute kidney disease. J Thorac Cardiovasc Surg. 2021;161(2):681–688.e3. doi: 10.1016/j.jtcvs.2019.10.101. 31959433

[6] Huen SC, Parikh CR. Predicting acute kidney injury after cardiac surgery: a systematic review. Ann Thorac Surg. 2012;93(1):337–347. doi: 10.1016/j.athoracsur.2011.09.010. 22186469 PMC3286599

[7] Cheruku SR, Raphael J, Neyra JA, et al. Acute kidney injury after cardiac surgery: prediction, prevention, and management. Anesthesiology. 2023;139(6):880–898. doi: 10.1097/ALN.0000000000004734. 37812758 PMC10841304

[8] Hu J, Chen R, Liu S, et al. Global incidence and outcomes of adult patients with acute kidney injury after cardiac surgery: a systematic review and meta-analysis. J Cardiothorac Vasc Anesth. 2016;30(1):82–89. doi: 10.1053/j.jvca.2015.06.017. 26482484

[9] Roh GU, Lee JW, Nam SB, et al. Incidence and risk factors of acute kidney injury after thoracic aortic surgery for acute dissection. Ann Thorac Surg. 2012;94(3):766–771. doi: 10.1016/j.athoracsur.2012.04.057. 22727320

[10] Thakar CV, Arrigain S, Worley S, et al. A clinical score to predict acute renal failure after cardiac surgery. J Am Soc Nephrol. 2005;16(1):162–168. doi: 10.1681/ASN.2004040331. 15563569

[11] Mehta RH, Grab JD, O’Brien SM, et al. Bedside tool for predicting the risk of postoperative dialysis in patients undergoing cardiac surgery. Circulation. 2006;114(21):2208–2216; quiz 2208. doi: 10.1161/CIRCULATIONAHA.106.635573. 17088458

[12] Wijeysundera DN, Karkouti K, Dupuis JY, et al. Derivation and validation of a simplified predictive index for renal replacement therapy after cardiac surgery. JAMA. 2007;297(16):1801–1809. doi: 10.1001/jama.297.16.1801. 17456822

[13] College of Intensive Care Medicine of Australia and New Zealand. Minimum standards for intensive care units. [cited 2024 Nov 20]. Available from: https://cicm.org.au/common/Uploaded%20files/Assets/Professional%20Documents/IC-1-Minimum-Standards-for-Intensive-Care-Units.pdf

[14] Ling L, Ho CM, Ng PY, et al. Characteristics and outcomes of patients admitted to adult intensive care units in Hong Kong: a population retrospective cohort study from 2008 to 2018. J Intensive Care. 2021;9(1):2. doi: 10.1186/s40560-020-00513-9. 33407925 PMC7788755

[15] STARRT-AKI Investigators, Canadian Critical Care Trials Group, Australian and New Zealand Intensive Care Society Clinical Trials Group, et al. Timing of initiation of renal-replacement therapy in acute kidney injury. N Engl J Med. 2020;383(3):240–251. doi: 10.1056/NEJMoa2000741. 32668114

[16] Crawford TC, Magruder JT, Grimm JC, et al. Renal failure after cardiac operations: not all acute kidney injury is the same. Ann Thorac Surg. 2017;104(3):760–766. doi: 10.1016/j.athoracsur.2017.01.019. 28434550

[17] Krauchuk A, Hrapkowicz T, Suwalski P, et al. Predictors of renal replacement therapy following isolated coronary artery surgery: a retrospective case–controlled study. Int J Surg. 2024;110(10):6684–6690. doi: 10.1097/JS9.0000000000001772. 38920325 PMC11487009

[18] Adriane P, Ardiyan A, Dewi NLK, et al. Predictors of renal replacement therapy in cardiac surgery-associated acute kidney injury patients: a single-centered, retrospective study. Bali J Anesthesiol. 2024;8(3):176–181. doi: 10.4103/bjoa.bjoa_149_24.

[19] Pickering JW, James MT, Palmer SC. Acute kidney injury and prognosis after cardiopulmonary bypass: a meta-analysis of cohort studies. Am J Kidney Dis. 2015;65(2):283–293. doi: 10.1053/j.ajkd.2014.09.008. 25445101

[20] Demirjian S, Huml A, Bakaeen F, et al. Sex bias in prediction and diagnosis of cardiac surgery associated acute kidney injury. BMC Nephrol. 2024;25(1):180. doi: 10.1186/s12882-024-03614-x. 38778259 PMC11112848

[21] Kulthinee S, Warhoover M, Puis L, et al. Cardiac surgery-associated acute kidney injury in cardiopulmonary bypass: a focus on sex differences and preventive strategies. Am J Physiol Renal Physiol. 2024;327(6):F994–F1004. doi: 10.1152/ajprenal.00106.2024. 39417779 PMC11687823

[22] Ko T, Higashitani M, Sato A, et al. Impact of acute kidney injury on early to long-term outcomes in patients who underwent surgery for type A acute aortic dissection. Am J Cardiol. 2015;116(3):463–468. doi: 10.1016/j.amjcard.2015.04.043. 26026862

[23] Wang Z, Ge M, Wang Z, et al. Identification of risk factors for postoperative stage 3 acute kidney injury in patients who received surgical repair for acute type A aortic dissection. BMC Surg. 2022;22(1):75. doi: 10.1186/s12893-022-01526-x. 35236329 PMC8892781

[24] Czerny M, Grabenwöger M, Berger T, et al. EACTS/STS guidelines for diagnosing and treating acute and chronic syndromes of the aortic organ. Eur J Cardiothorac Surg. 2024;65(2):ezad426. doi: 10.1093/ejcts/ezad426. 38408364

[25] Nishigawa K, Fukui T, Uemura K, et al. Preoperative renal malperfusion is an independent predictor for acute kidney injury and operative death but not associated with late mortality after surgery for acute type A aortic dissection. Eur J Cardiothorac Surg. 2020;58(2):302–308. doi: 10.1093/ejcts/ezaa063. 32182351

[26] Zhang K, Shang J, Chen Y, et al. The prognosis and risk factors for acute kidney injury in high-risk patients after surgery for type A aortic dissection in the ICU. J Thorac Dis. 2021;13(7):4427–4437. doi: 10.21037/jtd-21-823. 34422369 PMC8339792

[27] Geirsson A, Szeto WY, Pochettino A, et al. Significance of malperfusion syndromes prior to contemporary surgical repair for acute type A dissection: outcomes and need for additional revascularizations. Eur J Cardiothorac Surg. 2007;32(2):255–262. doi: 10.1016/j.ejcts.2007.04.012. 17500002

[28] Axtell AL, Fiedler AG, Melnitchouk S, et al. Correlation of cardiopulmonary bypass duration with acute renal failure after cardiac surgery. J Thorac Cardiovasc Surg. 2020;159(1):170–178.e2. doi: 10.1016/j.jtcvs.2019.01.072. 30826102

[29] Higo M, Shimizu Y, Wakabayashi K, et al. Post-operative kidney function using deep hypothermic circulatory arrest (DHCA) in aortic arch operation. Int J Nephrol Renovasc Dis. 2022;15:239–252. doi: 10.2147/IJNRD.S373828. 36189330 PMC9524279

[30] Englberger L, Suri RM, Li Z, et al. Validation of clinical scores predicting severe acute kidney injury after cardiac surgery. Am J Kidney Dis. 2010;56(4):623–631. doi: 10.1053/j.ajkd.2010.04.017. 20630639

[31] Kiers HD, van den Boogaard M, Schoenmakers MCJ, et al. Comparison and clinical suitability of eight prediction models for cardiac surgery-related acute kidney injury. Nephrol Dial Transplant. 2013;28(2):345–351. doi: 10.1093/ndt/gfs518. 23222415

[32] Zhang H, Yu M, Wang R, et al. Derivation and validation a risk model for acute kidney injury and subsequent adverse events after cardiac surgery: a multicenter cohort study. Int J Gen Med. 2022;15:7751–7760. doi: 10.2147/IJGM.S354821. 36249898 PMC9562825

[33] Jiang W, Xu J, Shen B, et al. Validation of four prediction scores for cardiac surgery-associated acute kidney injury in Chinese patients. Braz J Cardiovasc Surg. 2017;32(6):481–486. doi: 10.21470/1678-9741-2017-0116. 29267610 PMC5731314

[34] Loizzi F, Burattini O, Cafaro A, et al. Early acute kidney injury after transcatheter aortic valve implantation: predictive value of currently available risk scores. Hellenic J Cardiol. 2023;70:19–27. doi: 10.1016/j.hjc.2022.12.007. 36581137

[35] Palomba H, de Castro I, Neto ALC, et al. Acute kidney injury prediction following elective cardiac surgery: AKICS score. Kidney Int. 2007;72(5):624–631. doi: 10.1038/sj.ki.5002419. 17622275

[36] Prediction of acute kidney injury after cardiac surgery: model development using a Chinese electronic health record dataset. [cited 2025 Aug 24]. Available from: https://translational-medicine.biomedcentral.com/articles/10.1186/s12967-022-03351-5 10.1186/s12967-022-03351-5 PMC8994277 35397573

[37] Tian Y, Diao X, Wang Y, et al. Prediction scores for any-stage and stage-3 acute kidney injury after adult cardiac surgery in a Chinese population. J Cardiothorac Vasc Anesth. 2021;35(10):3001–3009. doi: 10.1053/j.jvca.2021.02.047. 33810934

[38] Godet G, Fleron MH, Vicaut E, et al. Risk factors for acute postoperative renal failure in thoracic or thoracoabdominal aortic surgery: a prospective study. Anesth Anal. 1997;85(6):1227–1232. doi: 10.1213/00000539-199712000-00009. 9390585

[39] Kim WH, Lee SM, Choi JW, et al. Simplified clinical risk score to predict acute kidney injury after aortic surgery. J Cardiothorac Vasc Anesth. 2013;27(6):1158–1166. doi: 10.1053/j.jvca.2013.04.007. 24050856

[40] Arnaoutakis GJ, Bihorac A, Martin TD, et al. RIFLE criteria for acute kidney injury in aortic arch surgery. J Thorac Cardiovasc Surg. 2007;134(6):1554–1561; discussion 1560–1561. doi: 10.1016/j.jtcvs.2007.08.039. 18023682

[41] Wang Z, Xu J, Zhang Y, et al. Prediction of acute kidney injury incidence following acute type A aortic dissection surgery with novel biomarkers: a prospective observational study. BMC Med. 2023;21(1):503. doi: 10.1186/s12916-023-03215-9. 38110934 PMC10729328

[42] Neyra JA, Hu MC, Minhajuddin A, et al. Kidney tubular damage and functional biomarkers in acute kidney injury following cardiac surgery. Kidney Int Rep. 2019;4(8):1131–1142. doi: 10.1016/j.ekir.2019.05.005. 31440703 PMC6698294

[43] Nezafati K, Cheruku S, Wanyan T, et al. Predicting in-hospital acute kidney injury after cardiac surgery using machine learning. J Cardiothorac Vasc Anesth. [cited 2025 Sep 23]; [S1053-0770(25)00814-6]. doi: 10.1053/j.jvca.2025.09.045. PMC13453055 41107168

[44] Members AF, Czerny M, Grabenwöger M, et al. EACTS/STS guidelines for diagnosing and treating acute and chronic syndromes of the aortic organ. Ann Thorac Surg. 2024;118(1):5–115. doi: 10.1016/j.athoracsur.2024.01.021. 38416090

[45] Ethgen O, Zarbock A, Koyner JL, et al. Early versus delayed initiation of renal replacement therapy in cardiac-surgery associated acute kidney injury: an economic perspective. J Crit Care. 2022;69:153977. doi: 10.1016/j.jcrc.2021.12.011. 35183893

[46] García-Fernández N, Pérez-Valdivieso JR, Bes-Rastrollo M, et al. Timing of renal replacement therapy after cardiac surgery: a retrospective multicenter Spanish cohort study. Blood Purif. 2011;32(2):104–111. doi: 10.1159/000324195. 21372568

[47] Valley TS, Nallamothu BK, Heung M, et al. Hospital variation in renal replacement therapy for sepsis in the United States. Crit Care Med. 2018;46(2):e158–e165. doi: 10.1097/CCM.0000000000002878. 29206766 PMC5771975

[48] Vaara ST, Serpa Neto A, Bellomo R, et al. Regional practice variation and outcomes in the standard versus accelerated initiation of renal replacement therapy in acute kidney injury (STARRT-AKI) trial: a post hoc secondary analysis. Crit Care Explor. 2024;6(2):e1053. doi: 10.1097/CCE.0000000000001053. 38380940 PMC10878545

[49] Privratsky JR, Krishnamoorthy V, Raghunathan K, et al. Postoperative acute kidney injury is associated with progression of chronic kidney disease independent of severity. Anesth Analg. 2022;134(1):49–58. doi: 10.1213/ANE.0000000000005702. 34908546

[50] Husain-Syed F, Ferrari F, Sharma A, et al. Persistent decrease of renal functional reserve in patients after cardiac surgery-associated acute kidney injury despite clinical recovery. Nephrol Dial Transplant. 2019;34(2):308–317. doi: 10.1093/ndt/gfy227. 30053231

[51] Legouis D, Galichon P, Bataille A, et al. Rapid occurrence of chronic kidney disease in patients experiencing reversible acute kidney injury after cardiac surgery. Anesthesiology. 2017;126(1):39–46. doi: 10.1097/ALN.0000000000001400. 27755064

