# Supplementary material for: Performance of Cleveland, Mehta, and Simplified Renal Index scores for predicting dialysis-requiring acute kidney injury after aortic vs. non-aortic cardiac surgery (2006–2023, 6160 patients)
Source: Ren Fail. 2025 Nov 26;47(1):2592437. doi: 10.1080/0886022X.2025.2592437 (PMC12667336; doi:10.1080/0886022X.2025.2592437)
Supplement: Additional_file_1_v3 - Clean file.docx [file IRNF_A_2592437_SM7382.docx]

# Supplemental material

# Supplementary Tables

**eTable 1.** Frequency of acute kidney injury requiring renal replacement therapy after cardiac surgery by risk category of Cleveland Clinic Score

**eTable 1A.** Entire cohort

**eTable 1B.** Aortic cohort

**eTable 1C.** Non-aortic cohort

**eTable 2.** Frequency of acute kidney injury requiring renal replacement therapy after cardiac surgery by risk category by risk category of the Mehta Score

**eTable 2A.** Entire cohort

**eTable 2B.** Aortic cohort

**eTable 2C.** Non-aortic cohort

**eTable 3.** Frequency of acute kidney injury requiring renal replacement therapy after cardiac surgery by risk category by risk category of the SRI

**eTable 3A.** Entire cohort

**eTable 3B.** Aortic cohort

**eTable 3C.** Non-aortic cohort

# Supplementary Figures

**eFigure 1.** Predictive performance of Cleveland Clinic Score for acute kidney injury requiring renal replacement therapy after cardiac surgery by risk category over time

**eFigure 2.** Predictive performance of Mehta Score for acute kidney injury requiring renal replacement therapy after cardiac surgery by risk category over time

**eFigure 3.** Predictive performance of Simplified Renal Index for acute kidney injury requiring renal replacement therapy after cardiac surgery by risk category over time

**Supplementary Table 1 – Frequency of acute kidney injury requiring renal replacement therapy after cardiac surgery by risk category** **by risk category of Cleveland Clinic Score**

**eTable 1A – Entire cohort**

| **Score** | **Risk Category** | **No. of counts (N=6160)** | **No. of RRT** | **% of RRT (95% CI)** |
| --- | --- | --- | --- | --- |
| 0-2 | Low | 3425 | 30 | 0.88% (0.6%-1.3%) |
| 3-5 | Intermediate | 2195 | 80 | 3.64% (2.9%-4.5%) |
| 6-8 | High | 468 | 69 | 14.74% (11.7%-18.4%) |
| ≥9 | Very high | 72 | 17 | 23.61% (14.7%-35.4%) |

**eTable 1B – Aortic cohort**

| **Score** | **Risk Category** | **No. of counts (N=1002)** | **No. of RRT** | **% of RRT (95% CI)** |
| --- | --- | --- | --- | --- |
| 0-2 | Low | 169 | 3 | 1.78% (0.5%-5.5%) |
| 3-5 | Intermediate | 559 | 26 | 4.65% (3.1%-6.8%) |
| 6-8 | High | 236 | 27 | 11.44% (7.8%-16.4%) |
| ≥9 | Very high | 38 | 11 | 28.95% (16.0%-46.1%) |

**eTable 1C – Non-aortic cohort**

| **Score** | **Risk Category** | **No. of counts (N=5158)** | **No. of RRT** | **% of RRT (95% CI)** |
| --- | --- | --- | --- | --- |
| 0-2 | Low | 3256 | 27 | 0.83% (0.6%-1.2%) |
| 3-5 | Intermediate | 1636 | 54 | 3.30% (2.5%-4.3%) |
| 6-8 | High | 232 | 42 | 18.10% (13.5%-23.8%) |
| ≥9 | Very high | 34 | 6 | 17.65% (7.4%-35.2%) |

**eTable 2 – Frequency of acute kidney injury requiring renal replacement therapy after cardiac surgery by risk category** **by risk category of the Mehta Score**

**eTable 2A – Entire cohort**

| **Score** | **No. of counts (N=6160)** | **No. of RRT** | **% of RRT (95% CI)** |
| --- | --- | --- | --- |
| 0-12 | 2601 | 35 | 1.35% (1.0%-1.9%) |
| 13-30 | 3418 | 122 | 3.57% (3.0%-4.3%) |
| 31-45 | 124 | 30 | 24.19% (17.2%-32.9%) |
| >45 | 17 | 9 | 52.94% (28.5%-76.1%) |

**eTable 2B – Aortic cohort**

| **Score** | **No. of counts (N=6160)** | **No. of RRT** | **% of RRT (95% CI)** |
| --- | --- | --- | --- |
| 0-12 | 466 | 16 | 3.43% (2.0%-5.6%) |
| 13-30 | 518 | 44 | 8.49% (6.3%-11.3%) |
| 31-45 | 17 | 6 | 35.29% (15.3%-61.4%) |
| >45 | 1 | 1 | 100% (5.5%-100%) |

**eTable 2C – Non-aortic cohort**

| **Score** | **No. of counts (N=6160)** | **No. of RRT** | **% of RRT (95% CI)** |
| --- | --- | --- | --- |
| 0-12 | 2135 | 19 | 0.89% (0.6%-1.4%) |
| 13-30 | 2900 | 78 | 2.69% (2.1%-3.4%) |
| 31-45 | 107 | 24 | 22.43% (15.2%-31.7%) |
| >45 | 16 | 8 | 50% (25.5%-74.5%) |

**eTable 3 – Frequency of acute kidney injury requiring renal replacement therapy after cardiac surgery by risk category** **by risk category of the SRI**

**eTable 3A – Entire cohort**

| **Score** | **Risk Category** | **No. of counts (N=6160)** | **No. of RRT** | **% of RRT (95% CI)** |
| --- | --- | --- | --- | --- |
| 0-1 | Low | 2566 | 17 | 0.66% (0.4%-1.1%) |
| 2-3 | Intermediate | 3169 | 114 | 3.60% (3.0%-4.3%) |
| ≥4 | High | 425 | 65 | 15.29% (12.1%-19.2%) |

**eTable 3B – Aortic cohort**

| **Score** | **Risk Category** | **No. of counts (N=1002)** | **No. of RRT** | **% of RRT (95% CI)** |
| --- | --- | --- | --- | --- |
| 0-1 | Low | 178 | 2 | 1.12% (0.2%-4.4%) |
| 2-3 | Intermediate | 734 | 47 | 6.40% (4.8%-8.5%) |
| ≥4 | High | 90 | 18 | 20.0% (12.6%-30.0%) |

**eTable 3C – Non-aortic cohort**

| **Score** | **Risk Category** | **No. of counts (N=5158)** | **No. of RRT** | **% of RRT (95% CI)** |
| --- | --- | --- | --- | --- |
| 0-1 | Low | 2388 | 15 | 0.63% (0.4%-1.1%) |
| 2-3 | Intermediate | 2435 | 67 | 2.75% (2.2%-3.5%) |
| ≥4 | High | 335 | 47 | 14.03% (10.6%-18.3%) |

**eTable 1 - eTable 3 Footnote**

Abbreviations: CI, confidence interval; RRT, renal replacement therapy

**Supplementary Figures**

**eFigure 1.** Predictive performance of Cleveland Clinic Score for acute kidney injury requiring renal replacement therapy after cardiac surgery by risk category over time


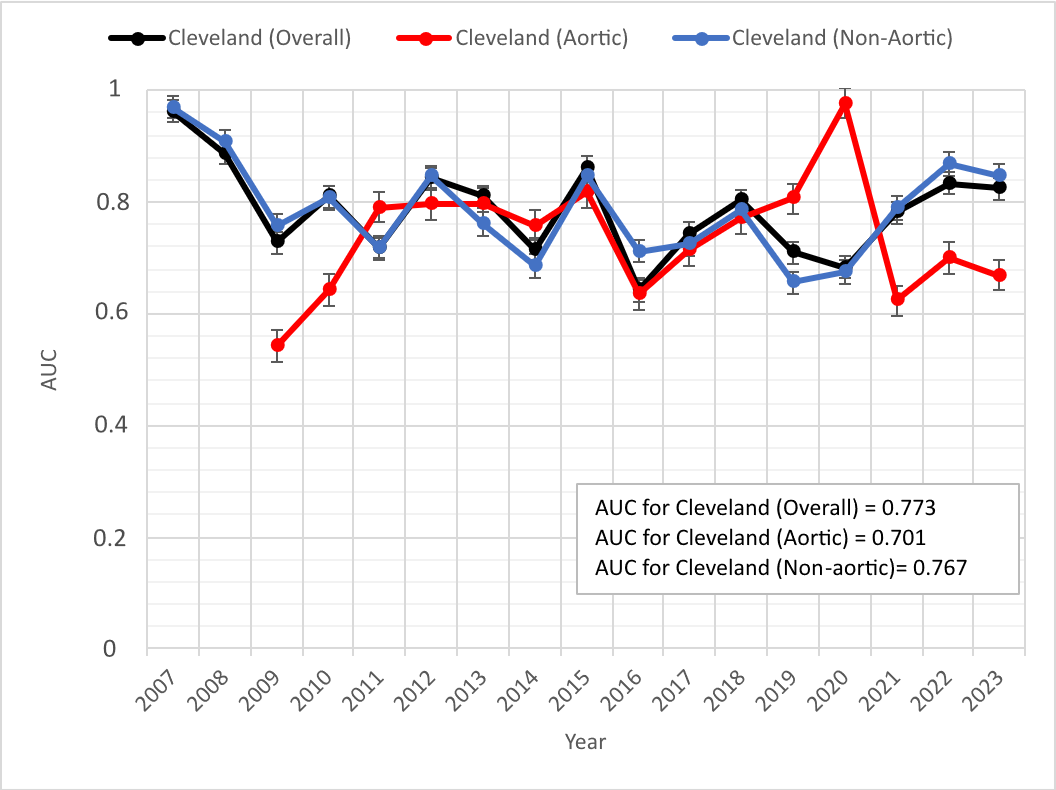


**eFigure 1 legend**

Area under receiver operating characteristic curves for discriminating acute kidney injury requiring renal replacement therapy after cardiac surgery by risk category of Cleveland Score over time from 2007 to 2023. Black line, overall cohort for aortic and non-aortic cardiac surgery. Red line, aortic surgery cohort. Blue line, non-aortic cardiac surgery cohort.

In the year 2006, there was no patient in either cohort with acute kidney injury requiring renal replacement therapy after cardiac surgery by risk category and therefore no AUC displayed.

In the years 2007 – 2008, there was no patient in the aortic surgery cohort with acute kidney injury requiring renal replacement therapy after cardiac surgery by risk category and therefore no AUC value is displayed.

Abbreviations: AUC, area under receiver operating curve.

**eFigure 2.** Predictive performance of Mehta Score for acute kidney injury requiring renal replacement therapy after cardiac surgery by risk category over time


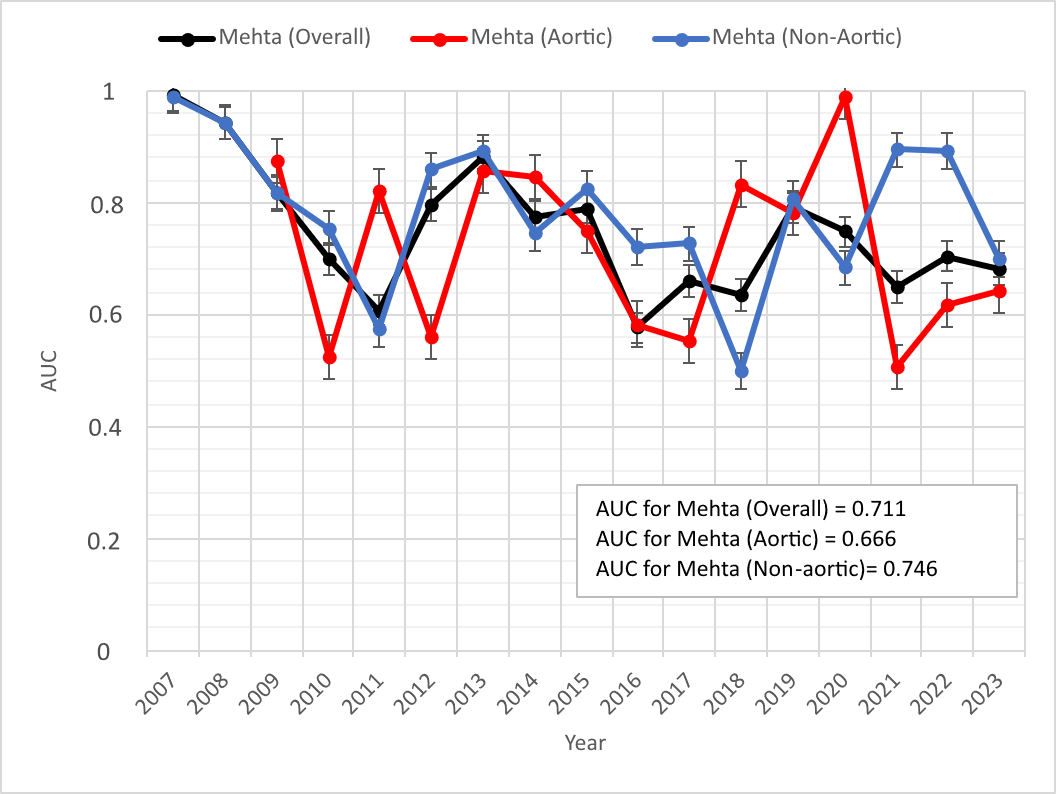


**eFigure 2 legend**

Area under receiver operating characteristic curves for discriminating acute kidney injury requiring renal replacement therapy after cardiac surgery by risk category of Mehta Score over time from 2007 to 2023. Black line, overall cohort for aortic and non-aortic cardiac surgery. Red line, aortic surgery cohort. Blue line, non-aortic cardiac surgery cohort.

In the year 2006, there was no patient in either cohort with acute kidney injury requiring renal replacement therapy after cardiac surgery by risk category and therefore no AUC displayed.

In the years 2007 – 2008, there was no patient in the aortic surgery cohort with acute kidney injury requiring renal replacement therapy after cardiac surgery by risk category and therefore no AUC value is displayed.

Abbreviations: AUC, area under receiver operating curve.

**eFigure 3.** Predictive performance of Simplified Renal Index for acute kidney injury requiring renal replacement therapy after cardiac surgery by risk category over time


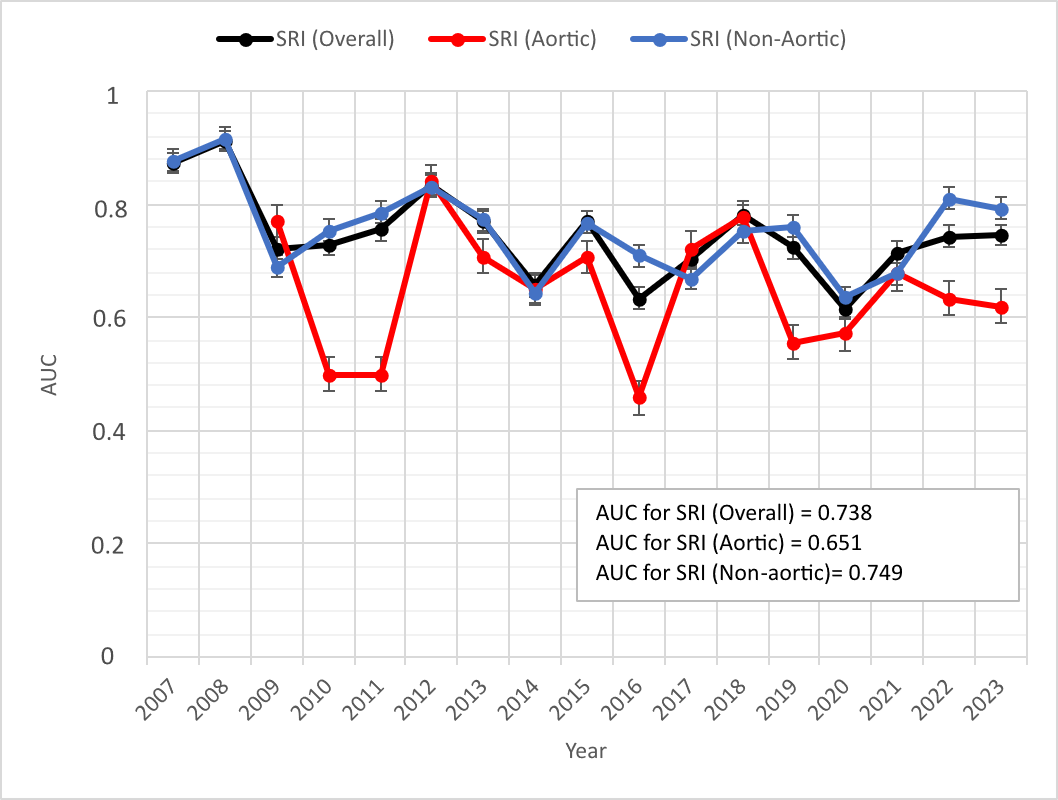


**eFigure 3 legend**

Area under receiver operating characteristic curves for discriminating acute kidney injury requiring renal replacement therapy after cardiac surgery by risk category of Simplified Renal Index over time from 2007 to 2023. Black line, overall cohort for aortic and non-aortic cardiac surgery. Red line, aortic surgery cohort. Blue line, non-aortic cardiac surgery cohort.

In the year 2006, there was no patient in either cohort with acute kidney injury requiring renal replacement therapy after cardiac surgery by risk category and therefore no AUC displayed.

In the years 2007 – 2008, there was no patient in the aortic surgery cohort with acute kidney injury requiring renal replacement therapy after cardiac surgery by risk category and therefore no AUC value is displayed.

Abbreviations: AUC, area under receiver operating curve.
